# Lower doses of dacarbazine (modified BEACODD) as a safer strategy with equal effectiveness in an intensive treatment protocol of Hodgkin's lymphoma: a preliminary retrospective analysis of a single public center in Brazil

**DOI:** 10.1016/j.htct.2024.06.003

**Published:** 2024-09-07

**Authors:** Larissa Hilario Dulley, Arthur Gomes Oliveira Braga, Guilherme Garcia Rodrigues, Sergio Costa Fortier, Carlos Sérgio Chiattone, Talita Maira Bueno da Silveira

**Affiliations:** a Irmandade de Santa Casa de Misericórdia de São Paulo, São Paulo, SP, Brazil; b A.C. Camargo Cancer Center, São Paulo, SP, Brazil

**Keywords:** Hodgkin lymphoma, Advanced disease, Dacarbazine

## Abstract

The German Hodgkin Study Group developed the escalated BEACOPP (bleomycin, etoposide, doxorubicin, cyclophosphamide, vincristine, procarbazine, and prednisone) protocol as a treatment strategy for advanced-stage Hodgkin's lymphoma. In Brazil, as well as in other countries, procarbazine has been replaced with dacarbazine due to the limited availability of procarbazine. The Hematology Center at Irmandade da Santa Casa de Misericórdia in São Paulo adopted and modified the escalated BEACOPP protocol, substituting prednisone with dexamethasone and incorporating two different doses of dacarbazine: 375 mg/m^2^/day on Day 8 or the original dose of 250 mg/m^2^/day on Days 2 and 3. This adjustment was made in response to the anticipated toxicity profile. This study aimed to compare the two different doses in the protocols (375 mg/m^2^/cycle versus 500 mg/m^2^/cycle) administered to patients with advanced Hodgkin's lymphoma in similar periods. This retrospective study analyzed the data of 31 patients at a single center in Brazil from 2019 to 2021. Seventeen of the 31 patients received 500 mg/m^2^/cycle (500 Group), while 14 received 375 mg/m^2^/cycle (375 Group). At the end of the protocol, 71% of the patients in the 375 Group and 76% in the 500 Group achieved complete remission. On analyzing the number of cycles that patients presented with febrile neutropenia, the 500 Group had three times more events (17.9%) than the 375 Group (6.09% - p-value = 0.04). In the 500 Group, 47.1% needed to change the protocol to ABVD (doxorubicin hydrochloride, bleomycin sulfate, vinblastine sulfate, and dacarbazine) due to toxicity. In this limited cohort from a single public center in Brazil, the use of 375 mg/m^2^ of dacarbazine per cycle of the modified escalated BEACOPP protocol emerged as a safer strategy, maintaining treatment efficacy without compromising response in patients with advanced Hodgkin's lymphoma.

## Introduction

Hodgkin's lymphoma (HL) is a hematologic neoplasm characterized by the presence of Reed-Sternberg cells within an inflammatory context of lymphoid tissues.^1^^,^^2^ HL accounts for over 12% of all lymphomas and is more commonly observed in individuals with two distinct age-related peaks: one between 20 and 30 years and the other ≥50 years old. It has a higher incidence in men and is more prevalent among individuals of Caucasian descent.^1^^,^^3^

The treatment of HL involves different strategies according to staging: early stage with favorable prognostic features (Ann Arbor Stage I), early stage with poor prognostic features (Stage II), and advanced stage disease (Stage III or IV).^1^^,^^2^ Generally, patients with advanced-stage disease undergo an extended course of chemotherapy without mandatory radiotherapy.^3^

The International Prognostic Score (IPS) is used to assess the prognosis of patients with HL. Generally, HL exhibits a favorable progression, with a survival rate of 85% or higher even though many patients with high-risk IPS (score of 3 or more) often fail to achieve the expected outcomes due to treatment-related toxicities and the aggressive nature of the lymphoma.^1^^,^^4^

To improve the outcomes of patients with advanced-stage disease, the BEACOPP (bleomycin, etoposide, doxorubicin, cyclophosphamide, vincristine, procarbazine, and prednisone) protocol was introduced in 1992 by the German Hodgkin Study Group. This treatment protocol aimed at increasing the dose intensity and decreasing treatment duration compared to the ABVD (doxorubicin hydrochloride, bleomycin sulfate, vinblastine sulfate, and dacarbazine) protocol by substituting etoposide with vinblastine and procarbazine.^5^

The effectiveness of the BEACOPP protocol was demonstrated in the HD9 Trial published in 2003. This trial compared BEACOPP, escalated BEACOPP, and COPP-ABV (COPP - Cyclophosphamide, Oncovin, Procarbazine and Prednisone; ABV - doxorubicin hydrochloride, bleomycin sulfate, and vinblastine sulfate). Despite being associated with significant toxicity, including thrombocytopenia (70%), leukopenia (90%), and infection (22%), BEACOPP has exhibited higher survival rates and less refractoriness.^5^

The German protocol involves the use of procarbazine, an alkylating agent not widely available in many countries, making its prescription challenging. In light of the EuroNet-PHL-C1 trial results, procarbazine was replaced with dacarbazine to maintain the COPP regimen and reduce toxicity. Consequently, other countries, such as Brazil, have adopted this modified protocol known as the escalated BEACODD (eBEACODD) protocol, which involves substituting prednisone with dexamethasone.^5^^,^^6^

In the Irmandade de Santa Casa Institution of São Paulo, the eBEACODD regimen was predominantly administered with 375 mg/m^2^ of dacarbazine on Day 8 (modified eBEACODD), instead of 250 mg/m^2^ on Days 2 and 3 (total: 500 mg/m^2^) as in the original trial. This dose reduction was prompted by reports of previous studies indicating elevated toxicity and poor tolerance to treatments with high doses of dacarbazine.

Thus, the present study retrospectively compared the efficacy and toxicity between two groups of patients from a single center in Brazil with advanced HL and IPS greater than or equal to 3. These groups were subjected to treatment with the eBEACODD regimen, with one group receiving 375 mg/m^2^/cycle (375 Group) and the other receiving 500 mg/m^2^/cycle (500 Group) of dacarbazine.

## Methods

### Study design and study population

This observational, retrospective, analytical, and descriptive study was conducted by evaluating the medical records of patients diagnosed with HL undergoing follow-up at a specialized oncological center in Brazil between 2019 and 2021, who were treated with the eBEACODD protocol. The patients were divided into two groups according to the dacarbazine doses used in the treatment protocol: one group was treated with 250 mg/m^2^/day on Days 2 and 3 (total of 500 mg/m^2^/cycle) and the other group was treated with 375 mg/m^2^/cycle on Day 8. The exclusion criterion was non-compliance with the treatment protocol.

### Statistical analysis

Thirty-one eligible patients were included in the study. The variables evaluated were sex, age, staging, number of cycles performed with each dacarbazine dose, and the number of episodes of febrile neutropenia in each cycle by group. Descriptive analysis of qualitative variables was performed using absolute and relative frequencies in addition to means, standard deviation, medians, minimum, and maximum values. Chi-square and Fisher's exact tests were employed to evaluate other variables. The significance level adopted was 5%. Statistical analyses were performed using IBM Statistical Package for Social Sciences (SPSS) software version 25.

## Results

Thirty-one patients were analyzed and divided into two groups: 17 in the 500 Group and 14 in the 375 Group. The baseline characteristics of the study population are presented in Table 1. Sixteen (51.6%) patients were men and 15 (48.4%) were women. Approximately 54.8% of the patients had advanced-stage disease (stage III and IV), while 51.6% had an IPS of ≥3. The median age was 30 years and the median number of nodal sites was 3.

**Table 1:** Baseline characteristics of the study population.

	All patients *n* = 31	500 mg/m^2^ *n* = 17	375 mg/m^2^ *n* = 14	p-value
Age (years) - n (range)	30 (14–47)	30 (16–47)	29.5 (14–41)	0.468
Gender - n (%)				0.843
Male	16 (51.6)	8 (47.1)	8 (57.1)	
Female	15 (48.4)	9 (52.9)	6 (42.9)	
Albumin (g/dL) - n (range)	3.0 (2–4)	3.0 (2–4)	2.0 (2–4)	**0.046**
Ann Arbor - n (%)				>0.99
Stages I-III	14 (45.2)	8 (47.1)	6 (42.9)	
Stage IV	17 (54.8)	9 (52.9)	8 (57.1)	
Leukocytes (/µL x 10^3^) - n (range)	10.6 (3.9–41.8)	10.6 (5.6–41.8)	10.75 (3.9–41.8)	0.811
Monocytes (/µL x 10^3^) - n (range)	1.24 (0.386–7.6)	1.63 (0.574–7.6)	0.84 (0.386–4.0)	0.230
Lymphocytes (/µL × 10^3^) - n (range)	1.59 (0.014–7.6)	1.54 (0.014–7.6)	1.88 (0.318–2.5)	>0.99
IPS score - n (%)				>0.99
<3	15 (48.4)	8 (47.1)	7 (50.0)	
≥3	16 (51.6)	9 (52.9)	7 (50.0)	
B symptoms - n (%)				0.344
Absent	5 (16.1)	4 (23.5)	1 (7.1)	
Present	26 (83.9)	13 (76.5)	13 (92.9)	
Fever (>38 °C) - n (%)				>0.99
No	16 (51.6)	9 (52.9)	7 (50.0)	
Yes	15 (48.4)	8 (47.1)	7 (50.0)	
Weight loss (>10%) - n (%)				**0.057**
No	20 (64.5)	8 (47.1)	12 (85.7)	
Yes	11 (35.5)	9 (52.9)	2 (14.3)	
Night sweats - n (%)				>0.99
No	20 (64.5)	11 (64.7)	9 (64.3)	
Yes	11 (35.5)	6 (35.3)	5 (35.7)	
Nodal sites	4 (1–4)	3 (1–4)	4 (2–4)	0.769
C-reactive protein (mg/dL) - n (range)	8.5 (1–95)	16 (1–95)	2 (1–19)	0.064

The 375 and 500 Groups had similar patient distributions, with variations in initial symptoms such as weight loss and C-reactive protein (CRP) levels. In the 375 Group, 85.7% did not report weight loss at the time of diagnosis, while 52.9% of the 500 Group reported weight loss. In terms of CRP results, the 500 Group had a median of 16 mg/dL, while the 375 Group had a median of 2 mg/dL. The median total white blood cell counts were 10,600/µL in the 500 Group and 10,750/µL in the 375 Group, with similar lymphocyte counts (total median: 1240/µL).

Table 2 presents the variables related to the treatment used, adverse effects such as related toxicities, and the necessity for de-escalation to the ABVD regimen, in addition to the response to treatment. Eight (25.8%) patients required de-escalation to ABVD due to toxicity, all of whom belong to the 500 Group.

**Table 2:** Outcomes.

	All patients *n* = 31	500 mg/m^2^ *n* = 17	375 mg/m^2^ *n* = 14	p-value
De-escalation to ABVD - n (%):				**0.003**
No	23 (74.2)	9 (52.9)	14 (100)	
Yes	8 (25.8)	8 (47.1)	0 (0)	
First line response - n (%):				>0.84
No information	5 (16.1)	3 (17.6)	2 (14.3)	
Complete remission	23 (74.2)	13 (76.5)	10 (71.4)	
Refractory disease	3 (9.7)	1 (5.9)	2 (14.3)	
Relapse or progression - n (%):				>0.99
No	27 (87.1)	15 (88.2)	12 (85.7)	
Yes	4 (12.9)	2 (11.8)	2 (14.3)	
Febrile neutropenia - n (%):				>0.99
No	17 (54.8)	9 (52.9)	8 (57.1)	
Yes	14 (45.2)	8 (47.1)	6 (42.9)	
Hospitalization for febrile neutropenia - n (%):				0.786
No	18 (58.1)	9 (52.9)	9 (64.3)	
Yes	13 (41.9)	8 (47.1)	5 (35.7)	
Number of cycles with a diagnosis of febrile neutropenia - n (%):				0.004
	19 (11.8)	14 (17.9)	5 (6.1)	

Complete response (CR) rates were comparable between the two groups, with 76.5% in the 500 Group and 71.4% in the 375 Group, as were the rates of disease progression or relapse, maintaining values of 11.8% and 14.3%, respectively.

A similar number of patients in both groups received the diagnosis of febrile neutropenia during treatment. However, when the percentages of patients hospitalized owing to this condition and the number of cases per cycle for each group were analyzed, an indication of increased toxicity was noted in the 500 Group, with 47.1% of the patients requiring hospitalization and 78 patients with febrile neutropenia receiving 14 cycles (17.9%).

In the statistical analysis, when the groups were compared, only the albumin levels and weight loss were statistically significant (p-value = 0.046 and 0.057, respectively). In terms of outcomes, only the number of patients who underwent ABVD de-escalation was significant (p-value = 0.003).

## Discussion

HL has established treatments supported by clinical trials and real-life studies, depending on its initial staging. However, in underdeveloped countries, such as Brazil, where access to all available treatments is not universally free, options become limited, especially within public services.^7^

In the context of advanced-stage HL, the optimal protocol in the public service remains under debate. The treatment would ideally combine greater effectiveness with minimal toxicity, but such a treatment has not yet been established.^7^^,^^8^

The BEACOPP protocol is one of the latest treatment regimens recognized as the gold standard for HL. It is acknowledged for its high toxicity compared with ABVD, but is associated with a lower incidence of refractoriness and relapse in advanced-stage diseases.^8^, ^9^, ^10^

As previously mentioned, the BEACOPP protocol is applicable in Brazil and other countries without access to procarbazine while maintaining its effectiveness. However, modifications were necessary, such as substituting procarbazine with dacarbazine. Notably, no studies have been conducted on the BEACODD protocol in Brazil to date.^8^^,^^11^

This retrospective study involved a retrospective assessment of the modified eBEACODD protocol, utilizing two different doses of dacarbazine. This protocol aimed at evaluating the toxicity of the treatment and exploring the potential of employing a lower dacarbazine dose while maintaining comparable efficacy in terms of response.

Initially, at Santa Casa de São Paulo, the treatment was administered at a dose of 375 mg/m^2^/cycle due to a history of elevated toxicity associated with other treatments. However, upon the publication of the BEACODD study recommending a dose of 500 mg/m^2^/cycle, the decision was made to align with the original protocol.

A noticeable escalation in adverse effects emerged when the higher dose was used, such as febrile neutropenia, increased hospitalization requirements, and the necessity to de-escalate to the ABVD regimen. Thus, the present study was conducted to corroborate the institution's apprehensions regarding treatment toxicity and ascertain whether reducing the dacarbazine dose resulted in any compromise of efficacy.

Despite the retrospective nature of this study and the limited sample size, the findings indicate that 8 of the 17 participants in the 500 Group required treatment de-escalation, due to febrile neutropenia. One participant in this Group required admittance to the intensive care unit. Notably, the 375 Group managed to maintain the same response rate at the end of treatment. The observed toxicity may be due to an impaired bone marrow caused by the disease or chemotherapy toxicity.

## Conclusion

The administration of a dacarbazine dose of 500 mg/m^2^ resulted in increased toxicity, as evidenced by the higher incidence of treatment-related febrile neutropenia and the need for de-escalation due to toxicity only in patients receiving this higher dose. By contrast, a dose of 375 mg/m^2^ demonstrated the potential to sustain the effectiveness of the original protocol with reduced toxicity. Therefore, a study with a larger number of patients is necessary to better understand the consequences of reducing the dacarbazine dose in the treatment of HL.

## Conflicts of interest

The authors declare no conflicts of interest

## References

[1] Li W., Li W (2022). Leukemia.

[2] Connors J.M., Cozen W., Steidl C. (2020). Hodgkin lymphoma published correction appears in Nat Rev Dis Primers. Nat Rev Dis Primers.

[3] Brice P., de Kerviler E., Friedberg J.W. (2021). Classical Hodgkin lymphoma. Lancet.

[4] Hasenclever D., Diehl V. (1998). A prognostic score for advanced Hodgkin's disease. International prognostic factors project on advanced Hodgkin's disease. N Engl J Med.

[5] Cheson B. (2007). MD Is BEACOPP better than ABVD?. Curr Hematol Malig Rep.

[6] Mounier N., Brice P., Bologna S. (2014). ABVD (8 cycles) versus BEACOPP (4 escalated cycles ≥4 baseline): final results in stage III-IV low-risk Hodgkin lymphoma (IPS 0-2) of the LYSA H34 randomized trial. Ann Oncol.

[7] Massimo F., Luminari S., Iannitto E. (2009). ABVD Compared with BEACOPP compared with CEC for the initial treatment of patients with advanced Hodgkin's lymphoma: results from the HD2000 Gruppo Italiano per lo Studio dei Linfomi Trial. J Clin Oncol.

[8] Anna S., Sturgess K., Brice P., Osborne W., Kirit M. (2019). Procarbazine-free escalated BEACOPDAC in frontline therapy of advanced Hodgkin in lymphoma reduces red cell transfusion requirements and may shorten time to menstrual period recovery compared to escalated BEACOPP and appears to be efficacious. Blood.

[9] Bröckelmann P., Eichenauer D., Jakob T., Follmann M., Engert A., Skoetz N. (2018). Hodgkin lymphoma in adults: diagnosis, treatment, and follow-up. Clinical Practice Guideline.

[10] Gautam A., Domenech E., Gautam A. (2019). Neutropenia during frontline treatment of advanced Hodgkin lymphoma: incidence, risk factors, and management. Crit Rev Oncol Hematol.

[11] Santarsieri A., Sturgess K., Brice P. (2021). Real world escalated beacopdac delivers similar outcomes to escalated BEACOPP and superior outcomes to response-adapted (RATHL) ABVD, while potentially reducing toxicity compared with escalated BEACOPP. Blood.

